# A Multicenter Retrospective Study on Clinical Characteristics and Outcome of Pyogenic Liver Abscess Focusing Multidrug-Resistant Organisms

**DOI:** 10.3390/jcm11041114

**Published:** 2022-02-19

**Authors:** Ji-Won Park, Jung-Hee Kim, Jang-Han Jung, Sung-Eun Kim, Hyoung-Su Kim, Haemin Jeong, Ki Tae Suk, Myoung-Kuk Jang, Dong-Joon Kim, Myung-Seok Lee, Sang-Hoon Park

**Affiliations:** 1 Department of Internal Medicine, Hallym University Sacred Heart Hospital of Hallym University Medical Center, 22, Gwanpyeong-ro 170 Beon-gil, Anyang-si 14068, Korea; miunorijw@hallym.or.kr (J.-W.P.); sekim@hallym.or.kr (S.-E.K.); 2 Institute for Liver and Digestive Diseases, Hallym University, Chuncheon-si 24252, Korea; hskim@kdh.or.kr (H.-S.K.); ktsuk@hallym.or.kr (K.T.S.); mkjang@kdh.or.kr (M.-K.J.); djkim@hallym.or.kr (D.-J.K.); 3 Department of Internal Medicine, Dongtan Sacred Heart Hospital of Hallym University Medical Center, Hwaseong-si 18450, Korea; jungheekim@hallym.or.kr (J.-H.K.); con2000@hallym.or.kr (J.-H.J.); 4 Department of Internal Medicine, Kangdong Sacred Heart Hospital of Hallym University Medical Center, 18, Cheonho-daero 173-gil, Seoul 05355, Korea; 5 Department of Internal Medicine, Chuncheon Sacred Heart Hospital of Hallym University Medical Center, 77, Chuncheon-si 24253, Korea; cromnjeong@hallym.or.kr; 6 Department of Internal Medicine, Kangnam Sacred Heart Hospital of Hallym University Medical Center, 1, Singil-ro, Seoul 07441, Korea; leemsmd@hallym.or.kr

**Keywords:** pyogenic liver abscess, multidrug-resistant organisms, extended-spectrum beta-lactamase

## Abstract

The emergence of multidrug-resistant organisms (MDROs) is a growing problem worldwide. However, little is known about the incidence, clinical features and outcomes of pyogenic liver abscesses (PLAs) caused by MDROs. A retrospective study of 833 patients with PLA admitted from 2008 to 2017 was performed. MDROs were found in 55 (6.6%) patients, and extended-spectrum beta-lactamase (ESBL)-producing Enterobacteriaceae was the most common causative microorganism. To evaluate the clinical features of and risk factors for MDRO-induced PLAs, propensity score matching (PSM) was performed in a 1:3 ratio (55 patients with MDROs and 165 patients without MDROs). After PSM, previous hepatobiliary procedure, preadmission exposure to antibiotics and elevated alkaline phosphatase levels were independent risk factors for MDRO-induced PLA. Sixteen patients (7.3%) died during hospitalization. Admission to intensive care unit (ICU), inadequate initial antibiotic treatment and use of inotropic agents were factors predictive of mortality. Although the presence of MDROs was not associated with in-hospital mortality, inadequate initial antibiotic treatment was prescribed to a large portion of the patients with MDRO-induced PLAs. We conclude that initial empirical antibiotic therapy for PLA should be based on the possibility of infection with MDROs, and close monitoring is necessary for patients with risk factors for in-hospital mortality.

## 1. Introduction

Pyogenic liver abscess (PLA), the most common type of visceral abscess, is a potentially life-threatening condition, with a mortality rate of up to 70% [1,2]. Although the advances in diagnostic techniques and therapeutic modalities have improved patient prognosis, the mortality rate remains within the range of 4–20% [3,4,5]. Previous studies have reported advanced age, the presence of a malignancy, biliary origin and high Acute Physiology and Chronic Health Evaluation II (APACHE II) scores as factors associated with a poor prognosis [4,5,6]. Most of these factors are related to host or clinical conditions rather than the pathogen. Liver abscesses may be caused by bacterial, fungal, or parasitic organisms. Prior to the 1980s, the most frequently isolated organism was *Escherichia coli (E. coli)* [7]. Since the first report of a liver abscess caused by *Klebsiella pneumoniae (K. pneumoniae)* leading to metastatic complications, such as endophthalmitis in Taiwan [8], a shift from *E.coli* to *K. pneumoniae* has occurred in Asia and some Western countries [9]. In general, antibiotic therapy composed of either a third-generation cephalosporin plus metronidazole or piperacillin/tazobactam has been empirically recommended for the treatment of PLA [10]. However, antibiotic resistance has become a major global public health problem, and some cases of PLA caused by extended-spectrum beta-lactamase (ESBL)-producing Enterobacteriaceae have been reported [11,12]. Multidrug-resistant organisms (MDROs) are defined as those with acquired nonsusceptibility to at least one agent in three or more antibiotic categories. The main types of MDROs include vancomycin-resistant enterococci (VRE), methicillin-resistant *Staphylococcus aureus* (MRSA), ESBL-producing Enterobacteriaceae and carbapenemase-producing Enterobacteriaceae. The emergence and rise of MDROs have become a major global public health concern [13]. Recently, Lo et al. reported an increasing trend in the prevalence of ESBL-producing *K. pneumoniae* in a series of Asian patients with PLA [14]. However, they did not investigate risk factors for ESBL-producing *K. pneumoniae* or the effect of MDROs on clinical outcomes. In Western countries, Mücke et al. focused on Enterococcus species as the main cause of PLA and reported the risk factors associated with VRE in a relatively small study population (86 patients in total) [15].

In this study, we investigated the clinical features and outcomes of PLA by reviewing clinical data collected over a 10-year period from patients in a large tertiary reference center in Korea. The primary endpoint was the identification of the risk factors for MDRO-induced PLA, and the secondary endpoint was the identification of the factors associated with in-hospital mortality.

## 2. Materials and Methods

### 2.1. Ethical Approval

Informed consent was waived because of the retrospective nature of the study and the analysis used anonymous clinical data, and this was approved by the ethics committee of Hallym University Medical Center. The study was conducted with the approval of the Institutional Review Board of Hallym University Medical Center (2019-06-007-001). All methods were carried out in accordance with relevant guidelines and regulations.

### 2.2. Study Population

This retrospective study was conducted in Korea at Hallym University Medical Center, which consists of six tertiary teaching hospitals, between January 2008 and December 2017. To identify suitable patients, the medical records of Hallym University Medical Center were systematically searched for code K75.0 of the International Classification of Disease, Tenth Revision. Patients with the following characteristics were included: (1) ultrasound (US), computed tomography (CT), or magnetic resonance imaging (MRI) findings compatible with a liver abscess and (2) either positive culture results from material retrieved from the abscess or the resolution of symptoms after antibiotic therapy. Patients with the following characteristics were excluded: (1) age < 18 years old and (2) parasitic/fungal/amoebic abscesses.

### 2.3. Clinical Data Collection and Definition

The medical records were systemically reviewed. Clinical information including sex, age, underlying medical conditions, initial symptoms, recent exposure to antibiotics and use of health care facilities within the 12 months before admission was gathered. Patients with a clinical picture indicative of cholecystitis, cholangitis, or documented bile duct disease were considered to have PLA secondary to biliary tract disease. Cryptogenic abscesses were diagnosed when there was no obvious source of infection after appropriate investigations. Initial laboratory values were defined as the values obtained on the first day of hospital admission or within 24 h after the clinical diagnosis of PLA, if PLA was not the initial cause of hospitalization. Blood cultures were performed for all patients. If patients were treated with percutaneous drainage or aspiration of the abscess, pus cultures were performed with the collected material. The percutaneous aspiration of the abscess cavity was performed with an 18- to 20-gauge needle under ultrasound guidance. For catheter drainage, catheters ranging in size from 7 to 12 French were introduced into the abscess cavity using the Seldinger technique. The abscess cavity was punctured with a sharp hollow needle under ultrasound guidance and fluoroscopy was used to confirm the position of the catheter. MDROs were considered present when strains showed nonsusceptibility to at least one agent in three or more classes of antimicrobial drugs. In-hospital mortality was defined as death during hospitalization.

### 2.4. Statistical Analysis

The statistical analysis was performed with SPSS version 24 (IBM Co., Armonk, NY, USA). Quantitative variables are expressed as the means ± standard deviations (SDs). A Student’s *t*-test was used to analyze continuous variables, and Fisher’s exact or Pearson’s chi-square tests were used to compare categorical variables. Factors that were significant in the univariate analysis were entered into a stepwise multivariate analysis to determine the independent risk factors before propensity score matching (PSM). Given the differences in baseline characteristics between patients with MDRO- and non-MDRO-induced PLA, PSM was used to enable the comparison of the variables of interest by matching participants based on confounding variables, such as age and comorbidities. Before PSM, our data showed a statistically significant difference in ‘age’ and ‘malignancy’ between MDRO and non-MDRO groups. The standardized difference was found to be greater than 0.1 in both variables; ‘age’ and ‘malignancy’. Therefore, we included ‘age’ and ‘malignancy’ in estimating the propensity score. Matching was performed in a 1:3 using the optimal matching method. After PSM, univariate analysis was performed to identify the factors associated with MDROs using Pearson’s chi-square or Fisher’s exact test analysis of variance. Variables significantly associated with MDROs in the univariate analysis were included in the multivariate logistic regression analysis, which was performed to identify the independent risk factors. Odds ratios and their 95% confidence intervals were calculated. All *p*-values < 0.05 were considered statistically significant and all *p*-values were two-tailed. Predictive factors for in-hospital mortality were analyzed with uni- and multivariate logistic regression models. Variables with *p* < 0.05 in the univariate model were entered into the multivariate stepwise logistic regression model (forward selection, likelihood ratio) to assess the independent predictive effect of the variables of interest.

## 3. Results

### 3.1. Baseline Characteristics of the Patients with PLA

A total of 833 patients with a diagnosis of PLA were eligible for inclusion in this study. The patients were divided into MDRO and non-MDRO groups only if the causative organisms and antibiotic susceptibility were identified by blood or pus culture. The baseline characteristics of the study subjects are provided in Table 1.

**Figure 1:**
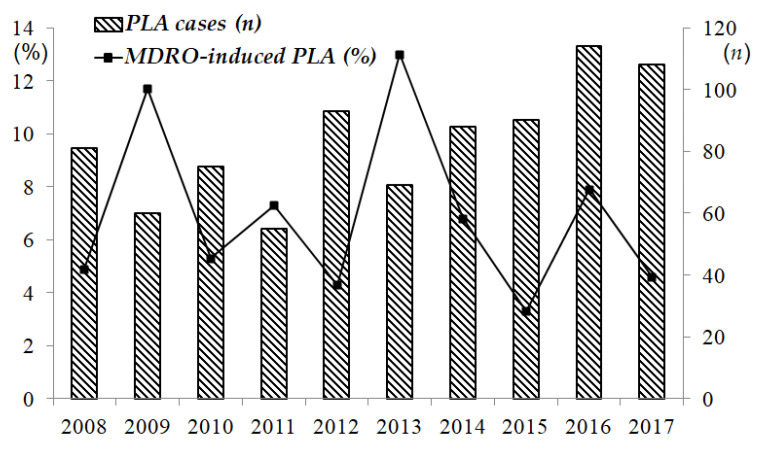
Pyogenic liver abscess incidence 2008–2017. The cases of pyogenic liver abscess have been increasing in recent years. Among liver abscess cases, the percentage of multidrug-resistant organism-induced pyogenic liver abscesses was highest in 2013 (13%) and lowest in 2015 (3.3%). The incidence rate fluctuated during this period. Note: PLA, pyogenic liver abscess, MDRO, multidrug-resistant organism.

During the past 10 years, the annual incidence of PLA ranged from 55 to 114 cases per year. On average, there were 9.9 cases (range from 7.0 to 11.8) per 100,000 admissions. The annual number of cases of PLA seemed to increase over the last 10 years, however, there was no statistical significance. Among the PLA cases, the percentage of MDRO-induced PLAs ranged from 3.3% to 13% per year, and the incidence rate showed a fluctuating pattern (Figure 1). The mean age of the patients was 62.2 years (±15.4; range 19 to 95), and most patients were male (536/833, 64.3%). The presumed etiologies of PLA were of cryptogenic origin (*n* = 623, 74.8%), biliary disorder (*n* = 180, 21.6%) and other infectious episodes, such as appendicitis, perforated diverticulitis or urinary tract infection (*n* = 30, 3.6%). In the study population, 26.2% (218/833) of the patients had type 2 diabetes mellitus (DM), and 15.7% (131/833) had malignancies. Fever (54.6%, 455/833) was the most common clinical manifestation, followed by abdominal pain (29.8%, 248/833). Radiologically, 66.4% (553/833) of the patients had solitary abscesses, and 63.3% (527/833) of the abscesses were located in the right lobe. The average size was 5.4 cm (±2.8; range from 0.3 to 17.6) (Table 1). Before PSM, compared with the clinical features of patients with PLAs caused by non-MDROs, the patients with PLAs caused by MDROs were older and tended to have a high rate of concomitant malignancies (Table 2).

### 3.2. Microbiological Investigations

**Figure 2:**
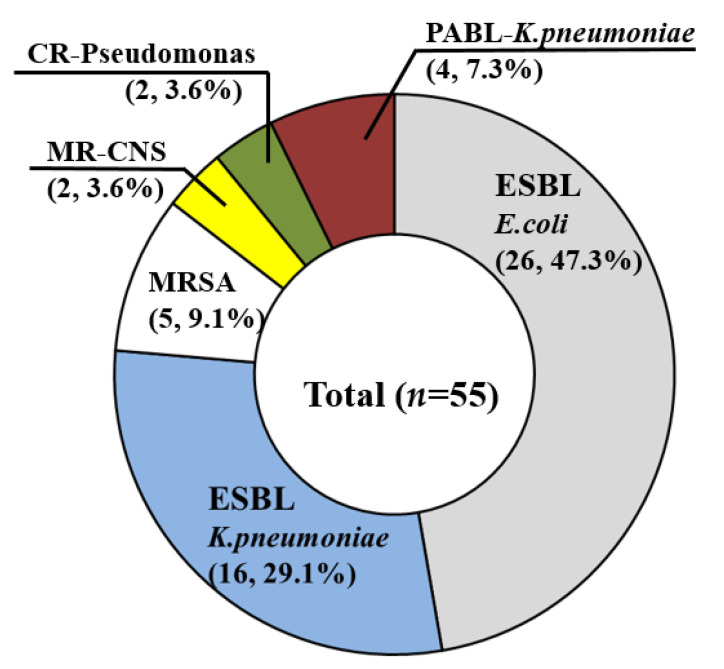
Types of multidrug-resistant organisms. Multidrug-resistant organisms were found in 55 patients. ESBL producing bacteria were the most common. ESBL-producing organisms consisted of *K. pneumoniae* (16/42, 38.1%) and *E.coli* (26/42, 61.9%). Note: ESBL, extended-spectrum beta-lactamase, MRSA, methicillin-resistant *Staphylococcus aureus*, MR-CNS, methicillin-resistant coagulase-negative Staphylococcus, CR, carbapenem-resistant, PABL, plasmid mediated AmpC β-lactamase.

Microbiological cultures of blood samples were performed for all 833 patients and were positive in 329 (39.5%). Pus cultures were performed for 728 patients who underwent aspiration or catheter drainage and were positive in 444 (61%). In 212 of the 833 patients (25.5%), both blood and pus cultures were positive. Among them, 198 patients (93.4%, 198/212) showed consistent results for blood and pus cultures. The most common organism identified in the blood and pus was *K. pneumoniae* (blood: 70.5%, pus: 73%), followed by *E. coli* (blood: 9.8%, pus: 9.7%). Of the other pathogens, Streptococcus species (blood: 17/63, 27%, pus: 27/72, 37.5%) and Enterobacter species (blood: 13/63, 20.6%, pus: 14/72, 19.4%) were frequently identified. Fifty-five patients (55/833, 6.6%) were diagnosed with PLAs caused by MDROs, and 506 patients were diagnosed with PLAs not caused by MDROs. Among the MDROs, ESBL-producing organisms were the most common, occurring in 42 out of the 55 cases (76.4%) (Figure 2). The ESBL-producing organisms were *K. pneumoniae* (16/42, 38.1%) and *E. coli* (26/42, 61.9%). In addition to ESBL-producing organisms, MRSA, plasmid-mediated AmpC β-lactamase (PABL)-producing organisms, carbapenem-resistant Pseudomonas and methicillin-resistant coagulase-negative Staphylococcus were identified in the MDRO group.

### 3.3. Characteristics of MDRO-Induced PLA after PSM

To evaluate the clinical features of and risk factors for MDRO-induced PLAs, PSM was performed in a 1:3 ratio. PSM yielded a cohort of 55 patients with MDROs and 165 patients without MDROs. After PSM, the differences in age and comorbidities between the two groups disappeared. Compared with the clinical features of patients with PLAs caused by non-MDROs, patients with PLAs caused by MDROs tended to have high alkaline phosphatase (ALP) levels and a low rate of ongoing alcohol use. In the group with PLAs caused by MDROs, many cases of PLA arose from biliary tract disease and prior hepatobiliary procedures (Table 3). Biliary causes included cholecystitis, cholangitis (mainly due to biliary stone), hepatopancreatobiliary malignancies, or others (periampullary duodenal diverticulum, choledochal cysts). Among those with prior hepatobiliary procedures, cholecystectomy, percutaneous transhepatic biliary drainage and endoscopic retrograde cholangiopancreatography (ERCP) were the most common procedures (Table 3). The rate of recent exposure to antibiotics or use of health care facilities within the 12 months before admission was significantly higher in the MDRO group.

When we compared the distribution of microorganisms between the MDRO and non-MDRO groups, *E. coli* was the predominant pathogen in the MDRO group. In contrast, in the non-MDRO group, *K. pneumoniae* was more common (Table 4). The location, size and number of PLAs on radiology were not significantly different between the MDRO and non-MDRO groups (Table 5). Multivariate stepwise logistic regression analyses showed that prior hepatobiliary procedures, recent exposure to antibiotics and elevated ALP levels were independent predictors of liver abscesses caused by MDROs (Table 6).

### 3.4. Treatment and Clinical Outcomes

**Figure 3:**
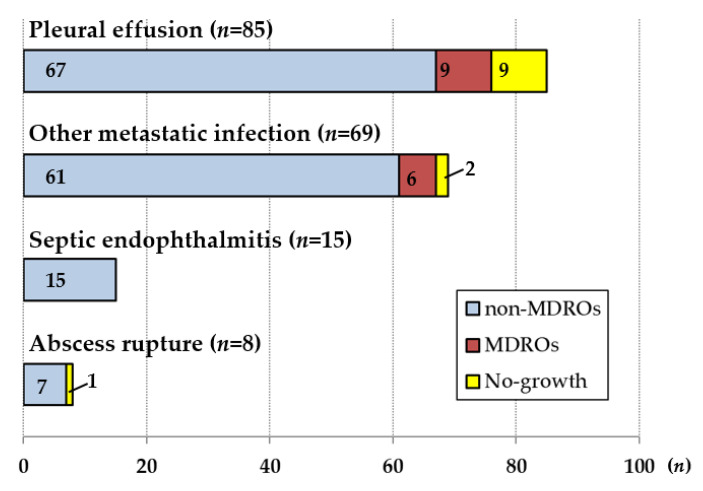
Abscess related complications. Pleural effusion was the most common complication. Infections at other sites included pneumonia, abscess formation in other organs, such as the musculoskeletal system or prostate, urinary tract infections, meningitis and infectious spondylitis. Note: MDROs, multidrug-resistant organisms.

All 833 patients with PLA were treated with antibiotics, and abscess drainage or aspiration was performed in 578 (69.4%). Patients were given third-generation cephalosporins (673/833, 80.8%), quinolones (51/833, 6.1%), piperacillin/tazobactam (46/833, 5.5%), carbapenem (42/833, 5%) or other drugs, such as fourth-generation cephalosporins or ampicillin/sulbactam (21/833, 2.5%) as the initial antibiotics. Twenty-seven patients were given a combination of antibiotics. Cefepime (fourth-generation cephalosporin) combined with amikacin was the most common regimen (8/27, 29.6%), and third-generation cephalosporin combined with intravenous metronidazole or quinolone was prescribed to 22.2% (6/27) and 18.5% (5/27) of the patients, respectively. In the MDRO group, patients were given third-generation cephalosporins (37/55, 67.3%), quinolones (4/55, 7.3%), piperacillin/tazobactam (4/55, 7.3%), carbapenem (5/55, 9.1%), or other drugs (5/55, 9.1%), and there were no significant differences between the MDRO and non-MDRO groups. In the MDRO group, two patients were prescribed cefepime (fourth-generation cephalosporin) combined with amikacin. The patients with PLAs caused by MDROs had significantly longer duration of intravenous antibiotic administration (29.5 ± 14.7 days vs. 22.1 ± 12.2 days). This is assumed to have influenced the duration of hospitalization, even though there were no significant differences between the two groups (28.5 ± 14.2 days vs. 24.8 ± 15.4 days). In 177 patients (177/833, 21.2%), abscess-related complications occurred (Figure 3). Pleural effusion was the most common complication (85/833, 10.2%). Septic endophthalmitis occurred in 15 patients (15/833, 1.8%). In addition to these complications, coexisting infections at other sites (69/833, 8.3%), and abscess rupture (8/833, 1%) were observed. Infections at other sites included pneumonia (31/833, 3.7%), abscess formation in other organs, such as the musculoskeletal system or prostate (31/833, 3.7%), urinary tract infections (2/833, 0.2%), meningitis (2/833, 0.2%) and infectious spondylitis (3/833, 0.4%). In the PSM analysis, the MDRO and non-MDRO groups had similar incidences of abscess-related complications. The frequencies of admission to the intensive care unit (ICU), surgical treatment and recurrence within 1 year tended to be higher in the MDRO group, but there were no significant differences (Table 5).

### 3.5. Factors Associated with in-Hospital Mortality

In total, thirty-four patients died during hospitalization, resulting in an overall mortality rate of 4.1% (34/833). In the PSM analysis, the in-hospital mortality rate was not significantly higher among patients with PLAs caused by MDROs than among those with non-MDRO-induced PLAs (12.7% vs. 5.5%, *p* = 0.072) (Table 5). In PSM analysis, we compared the clinical features between surviving (*n* = 204) and nonsurviving patients (*n* = 16) and found that nonsurviving patients had higher blood urea nitrogen (BUN) levels (≥20 mg/dL); were more likely to use inotropic agents; were more frequently admitted to ICU, and were more likely to have abscess-related complications. Furthermore, inadequate empirical antibiotic administration at admission was more common in the nonsurviving group. Multivariate analysis demonstrated that admission to ICU, inadequate empirical antibiotic treatment and use of inotropic agents were independently associated with in-hospital mortality (Table 7).

## 4. Discussion

In this study, we found that 6.6% (55/833) of the patients had PLAs caused by MDROs. ESBL- producing *K. pneumoniae* and *E.coli* accounted for 76.4% of the MDROs. Previously, Shi et al. found that among 817 patients with PLAs assessed from 2005 to 2015, 8.2% had PLAs caused by ESBL-producing Enterobacteriaceae isolates [16]. Our study showed that the number of PLA cases increased from 2008 to 2017 but that infection with MDROs has not increased in Korea. The incidence of ESBL-producing bacteria in our data appeared to be lower than that reported in a previous study (5% vs. 8.2%) [16]. In Western studies, the proportion of PLAs caused by antimicrobial-resistant organisms was reported to be 24.4–34.1% [15,17]. A recent study performed in the USA reported that there were no cases of infection with ESBL-producing *K. pneumoniae* [18]. These differences could be because microbes and resistance patterns show both regional and temporal variations.

In the present study, a previous history of hepatobiliary procedures, recent exposure to antibiotics and elevated ALP levels were risk factors for PLAs caused by MDROs. Due to a current lack of research focused on the difference between PLAs caused by MDROs and those not caused by MDROs, the characteristics of PLAs caused by MDROs have not been well described. Shi et al. found that the presence of biliary disorders, a history of treatment for malignancy, pulmonary infection at admission, and the absence of DM were associated with the occurrence of PLAs caused by ESBL-producing Enterobacteriaceae [16]. They reported that the mortality rates in patients with PLA caused by ESBL-producing Enterobacteriaceae were significantly higher than those in patients with PLA caused by non-ESBL-producing Enterobacteriaceae. However, the ESBL-producing group was older and more likely to have a history of treatment for malignancies than the non-ESBL-producing group. Similarly, our data before PSM showed that history of malignancies and advanced age were more common in the MDRO group. Advanced age and history of malignancy are already known factors predictive of a poor prognosis of PLAs and could influence mortality. Therefore, in our study, to correct this bias, PSM analysis was performed. In the present study, there was no relationship between MDRO-induced PLAs and abscess-related complications or DM. Instead, elevated ALP levels were found to be associated with MDRO-induced PLA. The serum ALP level is frequently elevated in a variety of liver diseases. A great many tissues, including the liver, the bones, the intestinal mucosa, and the placenta are particularly rich sources of the enzyme [19]. The increase in serum ALP levels is observed during cholestasis with increased bile acid concentration, due either to liver parenchymal damage or to biliary obstruction Abnormally high ALP level is considered the most reliable and consistent biochemical indicator of PLA. Although in our study, the ALP level had no relationship with the abscess size, previous studies reported that the ALP level was correlated with the abscess volume and disease duration [20,21]. According to a commonly held view, the reason for the prominent elevation in the level of ALP in the MDRO group was assumed to be the fact that the major cause of MDRO-induced PLAs was biliary disease. However, when we analyzed the clinical characteristics of MDRO-induced PLA only in patients with non-biliary causes, a significant increase in serum ALP levels was observed in the MDRO group (285.7 ± 175.9 vs. 197.2 ± 137.7, *p* = 0.005). Therefore, ALP elevation is likely to be related to the unrevealed pathologic condition of MDRO-induced PLA, further research on the mechanism of ALP elevation is required.

In the PSM analysis, the overall proportions of patients with malignancies were not different between the MDRO and non-MDRO groups. Although the presence of hepatopancreatobiliary malignancy was not a risk factor for MDRO-induced PLAs, empirical antibiotic treatment for an infection of unknown etiology, such as neutropenic fever following systemic chemotherapy or malignant biliary obstruction requiring repeated hepatobiliary procedures, might be related to the emergence of MDROs. In the present study, PLAs caused by MDROs had a significant relationship with prior hepatobiliary procedures, such as percutaneous transhepatic gallbladder drainage and/or ERCP. Sand et al. reported that ERCP, especially when performed with endoscopic sphincterotomy, may result in long-term bacterobilia [22]. Bacterobilia is an important factor in the pathogenesis of recurrent cholangitis and may be associated with gallstone formation, acute pancreatitis and cholangiocarcinogenesis [23,24,25]. Ultimately, the repeated administration of antibiotics to treat recurrent biliary tract infections can precipitate the emergence of MDROs by applying selective pressure.

The overall in-hospital mortality rate was 4.1%, which was slightly lower than previously reported rates (5–13%) [14,16,26,27], and the presence of MDRO alone was not an independent factor predictive of in-hospital mortality. In the present study, the initial administration of antibiotics that were later determined to be unsuitable based on the results of antibiotic susceptibility testing appeared to be associated with in-hospital mortality. Although the presence of MDROs was not associated with in-hospital mortality, inadequate initial antibiotic treatment was prescribed to a large portion of the patients with MDRO-induced PLAs. All patients except for 42 patients given carbapenem from the beginning received empirical antibiotics generally recommended for PLA treatment. In 42 patients given carbapenem as initial antibiotic treatment, only four patients were confirmed to be infected by ESBL-producing organisms, and twenty-two patients (52.4%, 22/42) were admitted to the ICU. This finding reflects that in clinical practice, admission to the ICU could be the main factor affecting the selection of carbapenem as an empirical antibiotic treatment. However, this is an inappropriate criterion. Therefore, accurate MDRO risk factor assessment is necessary to guide the selection of empirical antibiotics for the treatment of PLA.

This study had some limitations. First, this was a retrospective study based on the review of medical records. Therefore, previous antibiotic exposure and the use of prior healthcare facilities may have been underestimated in our study, and the patients lost to follow-up after hospital discharge may have affected the recurrence rate of PLA. Nevertheless, recent exposure to antibiotics was verified to be a factor predictive of MDRO-induced PLA, and the rate of recurrence of PLA also appeared to be similar to that in a previous study (4.5% vs. 5.3%) [28]. Second, the information was lacking: the types or duration of antibiotics or healthcare facilities used before admission, so a definite conclusion about these factors cannot be made. The information collected from medical records may inevitably have insufficiency that could impact the validity and attenuate the findings. To minimize bias and overcome the shortcomings of the retrospective study, we used PSM and subgroup analyses and found MDRO-related risk factors and prognostic factors associated with in-hospital mortality.

This was a large study involving more than 800 patients with PLA and one of the few studies to report the incidence and characteristics of MDRO-induced PLA. Although the present study sample is not representative of the distribution of patients with MDRO-induced PLAs worldwide, our data may be helpful for identifying patients in Asia who are at high risk of developing MDRO-induced PLA due to infection with the two leading causative pathogens, namely, *K. pneumoniae* and *E. coli*. In the future, a prospective study will be needed to discover more specific risk factors and create a MDRO-predictive model using risk factors in patients with PLA.

## 5. Conclusions

In conclusion, the patients with PLAs caused by MDROs had clinical features, such as a history of a hepatobiliary procedure, recent exposure to antibiotics and elevated ALP levels. Infection with MDROs was not a predictor of in-hospital mortality but was associated with a longer duration of treatment with intravenous antibiotics. For patients with PLAs with risk factors for in-hospital mortality and infection with MDROs, the initial use of broad-spectrum antibiotics suitable for the treatment of MDROs, followed by a reassessment of the treatment plan when culture results become available, might be an appropriate strategy.

## Figures and Tables

**Table 1:** Baseline Characteristics of the Study Population.

	Study Population (*n* = 833)	
Age	62.2 ± 15.4	
Sex (male, %)	536 (64.3%)	
Co-morbidities	Malignancy	131 (15.7%)
	Diabetes mellitus	218 (26.2%)
	Hypertension	306 (36.7%)
Ongoing alcohol use	247 (29.7%)	
Cause of abscess	Cryptogenic	623 (74.8%)
	Biliary cause	180 (21.6%)
	Other infectious cause	30 (3.6%)
Number of abscesses	Solitary	553 (66.4%)
	Multiple	280 (33.6%)
Size of abscess (cm)	5.4 ± 2.8	
Location of abscess	Right	527 (63.3%)
	Left	172 (20.6%)
	Both	134 (16.1%)
Initial empirical antibiotics	3rd-generation cephalosporins:ceftriaxone, cefotaxime	673 (80.8%)
	Quinolones: levofloxacin, ciprofloxacin	51 (6.1%)
	Piperacillin/tazobactam	46 (5.5%)
	Carbapenem: meropenem, ertapenem	42 (5%)
	Other drugs	21 (2.5%)

Data are presented as *n* (%) or mean ± standard deviation.

**Table 2:** Comparison of clinical features before propensity score matching.

Variables	MDROs (*n* = 55)	Non-MDROs (*n* = 506)	*p*-Value
Age (years)	68.4 ± 14.7	62.5 ± 14.2	0.004
Sex (male, %)	30 (54.5)	340 (67.2)	0.060
Malignancy	22 (40)	64 (12.7)	<0.001
Diabetes mellitus	15 (27.3)	140 (27.7)	0.950
Hypertension	23 (41.8)	200 (39.5)	0.741
Ongoing alcohol use	6 (10.9)	157 (31)	0.005
Cause of abscess			<0.001
Cryptogenic	27 (49.1)	395 (78.1)	
Biliary cause	28 (50.9)	95 (18.8)	
Other infectious cause	0	16 (3.2)	
Prior HB procedure	26 (47.3)	73 (14.4)	<0.001
Cholecystectomy	7 (26.9)	37 (50.7)	
Hepatic resection	2 (7.7)	1 (1.4)	
PTGBD or stent insertion	7 (26.9)	13 (17.8)	
ERCP	6 (23.1)	11 (15.1)	
Biliary tract operation	3 (11.5)	9 (12.3)	
TACE	1 (3.8)	2 (2.7)	
Recent exposure to antibiotics	34 (61.8)	137 (27.1)	<0.001
Recent prior hospitalization	30 (54.5)	108 (21.3)	<0.001
Laboratory finding			
WBC (×10^3^/μL)	12.2 ± 6.1	13.5 ± 6.9	0.154
C-reactive protein (mg/L)	172.0 ± 92.4	199.2 ± 91.9	0.038
Albumin (g/dL)	3.2 ± 0.5	3.3 ± 0.6	0.051
ALT (IU/L)	83.7 ± 125.5	91.9 ± 107.0	0.598
ALP (U/L)	328.6 ± 308.0	249.2 ± 223.7	0.017
Total bilirubin (mg/dL)	1.9 ± 1.9	1.7 ± 1.9	0.395
Hemoglobin A1c	6.3 ± 1.4	7.3 ± 2.2	0.003
BUN (mg/dL)	21.4 ± 10.7	22.1 ± 16.3	0.737
Creatinine (mg/dL)	1.0 ± 0.4	1.6 ± 5.1	0.637

Data are presented as *n* (%) or mean ± standard deviation. Abbreviations: MDROs: multidrug-resistant organisms; HB: hepatobiliary; PTGBD: percutaneous transhepatic gallbladder drainage; ERCP: endoscopic retrograde cholangiopancreatography; TACE: transcatheter arterial chemoembolization, WBC: white blood cell, ALT: alanine aminotransferase, ALP: alkaline phosphatase, BUN: blood urea nitrogen.

**Table 3:** Comparison of clinical features after propensity score matching.

Variables	MDROs (*n* = 55)	Non-MDROs (*n* = 165)	*p*-Value
Age (years)	68.4 ± 14.7	66.6 ± 15.2	0.455
Sex (male, %)	30 (54.5)	100 (60.6)	0.429
Malignancy	22 (40)	64 (38.8)	0.873
Diabetes mellitus	15 (27.3)	43 (26.1)	0.860
Hypertension	23 (41.8)	66 (40.0)	0.812
Ongoing alcohol use	6 (10.9)	49 (29.7)	0.005
Cause of abscess			0.006
Cryptogenic	27 (49.1)	112 (67.9)	
Biliary cause	28 (50.9)	47 (28.5)	
Benign biliary cause	15	19	
Malignant biliary cause	13	28	
Other infectious cause	0	6 (3.6)	
Prior HB procedure	26 (47.3)	39 (23.6)	0.001
Cholecystectomy	7 (26.9)	13 (33.3)	
Hepatic resection	2 (7.7)	1 (2.6)	
PTGBD or stent insertion	7 (26.9)	12 (30.8)	
ERCP	6 (23.1)	4 (10.3)	
Biliary tract operation	3 (11.5)	7 (17.9)	
TACE	1 (3.8)	2 (5.1)	
Recent exposure to antibiotics	34 (61.8)	54 (32.7)	<0.001
Recent prior hospitalization	30 (54.5)	50 (30.3)	0.001
Laboratory finding			
WBC (×10^3^/μL)	12.2 ± 6.1	13.5 ± 7.8	0.236
C-reactive protein (mg/L)	172.0 ± 92.4	193.2 ± 91.4	0.139
Albumin (g/dL)	3.2 ± 0.5	3.3 ± 0.6	0.087
ALT (IU/L)	83.7 ± 125.5	85.2 ± 105.0	0.931
ALP (U/L)	328.6 ± 308.0	236.2 ± 204.0	0.012
Total bilirubin (mg/dL)	1.9 ± 1.9	1.8 ± 2.2	0.643
Hemoglobin A1c	6.3 ± 1.4	6.9 ± 2.1	0.170
BUN (mg/dL)	21.4 ± 10.7	22.5 ± 15.2	0.604
Creatinine (mg/dL)	1.0 ± 0.4	1.2 ± 0.8	0.008

Data are presented as *n* (%) or mean ± standard deviation. Abbreviations: MDROs: multidrug-resistant organisms; HB: hepatobiliary; PTGBD: percutaneous transhepatic gallbladder drainage; ERCP: endoscopic retrograde cholangiopancreatography; TACE: transcatheter arterial chemoembolization, WBC: white blood cell, ALT: alanine aminotransferase, ALP: alkaline phosphatase, BUN: blood urea nitrogen.

**Table 4:** Comparison of microbiological results after propensity score matching.

Variables	MDROs (*n* = 55)	Non-MDROs (*n* = 165)	*p*-Value
Blood culture-total number of positive results	37	112	<0.001
*Klebsiella pneumoniae*	12 (32.4)	77 (68.8)	
*Escherichia coli*	18 (48.6)	8 (7.1)	
*K. pneumonia* & *E. coli*	0	1 (0.9)	
Other pathogens	7 (18.9)	26 (23.2)	
Pus culture-total number of positive results	39	120	<0.001
*Klebsiella pneumoniae*	16 (41.0)	81 (67.5)	
*Escherichia coli*	17 (43.6)	10 (8.3)	
*K. pneumonia* & *E. coli*	1 (2.6)	2 (1.7)	
Other pathogens	5 (12.8)	27 (22.5)	

Data are presented as number (%). Abbreviations: MDROs: multidrug-resistant organisms.

**Table 5:** Comparison of radiologic findings, treatment and clinical outcomes after propensity score matching.

Variables	MDROs (*n* = 55)	Non-MDROs (*n* = 165)	*p*-Value
Number of abscesses (Multiple)	24 (43.6)	61 (37.0)	0.379
Size of abscess (cm)	4.9 ± 2.7	5.5 ± 2.7	0.276
Location of abscess			0.107
Right	39 (70.9)	95 (57.6)	
Left	6 (10.9)	39 (23.6)	
Both	10 (18.2)	31 (18.8)	
Initial treatment			0.119
Antibiotics only	15 (27.3)	29 (17.6)	
Antibiotics with drainage	40 (72.7)	136 (82.4)	
Vasopressor or inotropic agent use	8 (14.5)	23 (13.9)	0.911
Surgical treatment	3 (5.5)	2 (1.2)	0.068
ICU care	20 (36.4)	44 (26.7)	0.170
Duration of antibiotics (day)	41.4 ± 19.0	44.1 ± 30.7	0.453
Intravenous antibiotics (day)	29.5 ± 14.7	21.5 ± 12.1	<0.001
Duration of hospitalization (day)	28.5 ± 14.2	24.1 ± 15.4	0.067
Complications	15 (27.3)	41 (24.8)	0.602
Septic endophthalmitis	0	3 (1.8)	
Other metastatic infection	6 (10.9)	15 (9.1)	
Pleural effusion	9 (16.4)	20 (12.1)	
Abscess rupture	0	3 (1.8)	
Recurrence within 1 year	4 (9.8)	8 (6.3)	0.463
Death	7 (12.7)	9 (5.5)	0.072

Data are presented as *n* (%) or mean ± standard deviation. Abbreviations: MDROs: multidrug-resistant organisms.

**Table 6:** Multivariate analysis of risk factors in relation to multidrug-resistant organisms-induced liver abscess.

Variables	Univariate Analysis	*p*-Value	Multivariate Analysis	*p*-Value
	Odds Ratio (95% CI)		Odds Ratio (95% CI)	
Ongoing alcohol use	0.290 (0.117–0.721)	0.008		
Cause of abscess (biliary vs. non-biliary cause)	2.604 (1.390–4.876)	0.003		
History of prior HB procedure	2.897 (1.528–5.492)	0.001	2.086 (1.050–4.144)	0.036
Recent exposure to antibiotics	3.328 (1.766–6.272)	<0.001	2.508 (1.281–4.909)	0.007
Recent prior hospitalization	2.760 (1.476–5.162)	0.001		
ALP, per log10 (U/L)	4.972 (1.817–13.605)	0.002	3.883 (1.362–11.068)	0.011

Abbreviations: CI: confidence interval; HB: hepatobiliary; ALP: alkaline phosphatase.

**Table 7:** Multivariate analysis of risk factors associated with in-hospital mortality.

Variables	Univariate Analysis	*p*-Value	Multivariate Analysis	*p*-Value
	Odds Ratio (95% CI)		Odds Ratio (95% CI)	
Old age (≥70)	2.843 (0.953–8.478)	0.061		
BUN (≥20 mg/dL)	3.955 (1.233–12.678)	0.021		
Inadequate antibiotics	3.511 (1.228–10.032)	0.019	3.472 (1.034–11.656)	0.044
Rupture of liver abscess	29 (2.476–339.709)	0.007		
Other metastatic infection	5.341 (1.651–17.276)	0.005		
Use of inotropic agents	14.524 (4.795–43.995)	<0.001	4.228 (1.142–15.654)	0.031
ICU admission	21.560 (4.736–98.140)	<0.001	9.586 (1.724–53.291)	0.010
MDRO-induced abscess	2.528 (0.894–7.147)	0.080		

Abbreviations: CI: confidence interval, BUN: blood urea nitrogen, ICU: intensive care unit, MDRO: multidrug-resistant organism.

## Data Availability

The data presented in this study are available on request from the corresponding author.
